# High-frequency ecological momentary assessment of emotional and interpersonal states preceding and following self-injury in female adolescents

**DOI:** 10.1007/s00787-020-01626-0

**Published:** 2020-08-29

**Authors:** Julian Koenig, Julia Klier, Peter Parzer, Philip Santangelo, Franz Resch, Ulrich Ebner-Priemer, Michael Kaess

**Affiliations:** 1 University Hospital of Child and Adolescent Psychiatry and Psychotherapy, University of Bern, Bern, Switzerland; 2 Section for Experimental Child and Adolescent Psychiatry, Department of Child and Adolescent Psychiatry, Centre for Psychosocial Medicine, University of Heidelberg, Heidelberg, Germany; 3 Clinic for Child and Adolescent Psychiatry, Centre for Psychosocial Medicine, University of Heidelberg, Heidelberg, Germany; 4 Chair of Applied Psychology/Mental mHealth Lab, Institute of Sport and Sports Science, Karlsruhe Institute of Technology, Karlsruhe, Germany; 5 Section for Translational Psychobiology in Child and Adolescent Psychiatry, Department of Child and Adolescent Psychiatry, Centre for Psychosocial Medicine, University of Heidelberg, Heidelberg, Germany

**Keywords:** Adolescents, Self-injury, Ecological momentary assessment, Negative affect

## Abstract

Non-suicidal self-injury (NSSI) is a considerable health problem among adolescents. Affect regulation by means of self-injury may promote the maintenance of NSSI. However, existing findings have limited ecological validity. The present study aimed to assess emotional and interpersonal states preceding and following incidents of NSSI in female adolescents. Adolescents with NSSI-disorder completed ecological momentary assessments of affective and interpersonal states on an hourly basis for multiple days. Multilevel mixed-effect regression analyses were conducted to assess antecedences and consequences of acts of self-injury. Data from *n* = 73 female adolescents covering a total of 52 acts of self-injury were available for analyses. The urge to self-injure on the between subject-level and negative affect on the within-level were best predictors of self-injury. Surprisingly, self-injury increased negative affect and decreased feelings of attachment (mother only) in the following hour. In line with findings in adults, results illustrate the important association between negative affect and self-injury in female adolescents. However, the occurrence of NSSI itself was related to concurrent increases in negative affect, and even prospectively predicted a consecutive increase in negative affect. Therefore, improvements of negative affect following (or during) self-injury, as previously reported, are at best short-lived (< 1 h).

## Introduction

Self-injurious behavior (i.e., cutting, burning) is a serious problem among adolescents. Non-suicidal self-injury (NSSI) has been introduced in the 5th version of the Statistical and Diagnostic Manual of Mental Disorders (DSM-5) under section 3, as a disorder warranting further research. NSSI often occurs in the context of psychiatric conditions (such as depression), and is considered a key feature of the borderline personality disorder (BPD). The prevalence for single events of NSSI in non-clinical samples according to a recent meta-analysis is 17.2% among adolescents, 13.4% among young adults, and 5.5% among adults [1]. The prevalence of NSSI disorder (according to DSM-5 diagnostic criteria) is estimated at 5% among non-clinical samples of adolescents, and rates between 50 and 80% among in-patient samples are reported [2–4]. Given its prevalence, the World Health Organization has recognized NSSI as one of the top five major health threats to adolescents. Previous NSSI increases the risk for future NSSI [5, 6] and it has repeatedly been shown that NSSI predicts future suicide attempts [5–9]. NSSI is associated with greater mortality [10] and increased risk for suicide, even 15 years later [11, 12].

Whereas reasons to engage in NSSI are manifold, functions of NSSI are best described as two-factorial, constituting of two conceptually distinguishable constructs: (1) intrapersonal functions (i.e., affect regulation), and (2) social functions (i.e., interpersonal influence) [13]. This two-factor structure has been found across samples (adolescents, young adults, adults) and settings (university, clinical), [14, 15] suggesting that it probably generalizes to diverse populations [13]. Based on self-report measures, NSSI is associated with difficulties in emotion regulation [16] and youth engaging in NSSI report greater emotion reactivity [17]. NSSI occurs during states of intense negative affect and—based on self-reports—is capable to (momentarily) reduce negative affect [18]. NSSI in adolescents is most frequently performed as dysfunctional strategy to regulate intense emotions, in particular to reduce negative affect [19]. For example, in a community sample of adolescents (*n* = 663, 57% female, 15.5 ± 1·18 years), including *n* = 293 adolescents endorsing NSSI in the past year (12.87 ± 29.4 incidents of self-injury in the past year), intra- and interpersonal functions, such as ‘to try to get a reaction from someone’, ‘to get control of a situation’, and ‘to stop bad feelings’, were most common reasons to engage in self-injury [20]. Decreased negative affect following acts of NSSI may promote maintenance of NSSI and has been linked to more life-time acts of NSSI [21].

Studies using structured interviews or self-reports to address the functions of self-injury support an affect-regulating function of self-injury. Studying a sample of *n* = 39 self-injurers (77% female, 19.4 ± 2.4 years, 17.2 ± 13.2 lifetime incidents of self-injury), Klonsky reported that participants indicated that self-injury was associated with improvements in affective valence and decreases in affective arousal. Participants reported to “feel overwhelmed, sad, and frustrated before self-injury, and relieved and calm after self-injury” [22]. Experimental studies, addressing changes in momentary affect following experimental pain induction, in patients with BPD or those engaging in self-injury, seem to confirm the affect regulating function of NSSI. Recently, we have shown that adolescents engaging in NSSI report improved mood after experimental stimulation with cold pain [23]. Findings are in line with neuroimaging research in adults with BPD, suggesting amygdala deactivation as neural mechanism underlying pain-mediated affect regulation [24–26].

Studies based on retrospective interviews or self-reports are limited to address changes in affective states preceding and following actual acts of self-injury. Similar, experimental laboratory-based studies have limited ecological validity, given the experimental setting and type of pain induction (i.e., experimenter based). The aim of the present study was to address changes in momentary affect and interpersonal attachment preceding and following actual incidents of self-injury in adolescents engaging in NSSI using Ecological Momentary Assessment (EMA). By enabling repeated assessments of momentary states, EMA is well suited to track symptom dynamics and within-person processes over time in everyday life. Accordingly, EMA is well suited to investigate unstable symptomatology, such as affective states [27–30].

Previous EMA studies in adults attempted to study predictors of self-injury based on affective states. However, frequently studies were limited by an insufficient sampling frequency. High-frequency EMA is capable to capture short-lived dynamics that may be missed by lower sampling frequencies (for an example see [31]). Ammermann et al. assessed levels of positive and negative affect in adults with BPD and comorbid depression using EMA across 7 days, with four daily assessments [32]. Andrewes et al. [33] used EMA to study motives of NSSI in a sample of youth (15–25 years) with BPD (*n* = 24; 52 actual NSSI events). Again, EMA was limited to six daily assessments across 6 days (also see [34]).

Other studies, like those by Anestis et al. [35, 36] investigated the course of NSSI incidents over 2 weeks in *n* = 127 (100% females, 25.34 ± 7.71 years) using EMA (6 prompts per day, semi-random). Armey et al. [37] studied *n* = 36 individuals (75% female, 18.70 ± 0.79 years) with lifetime NSSI using EMA (7 days, 6 random prompts per day, 10 am to 10 pm, *n* = 17 incidents). Detectable changes in affect were reported 8 h before NSSI, but the random-sampling strategy prohibited detailed analyses of affect immediately before and after actual NSSI. Bresin et al. [38] used EMA to study self-injury in a college-based sample of *n* = 67 (57% female, 19.58 ± 2.94 years) with lifetime self-injury (median 15 lifetime NSSI events, range 1–1000). EMA (14 days, 1 retrospective assessment of the day in the evening) was based on 74 events. Muehlenkamp et al. studied positive and negative affect in *n* = 131 (100% female, 25.3 ± 7.6 years) bulimia patients, including *n* = 19 reporting at least one event of NSSI during the EMA (14 days, event-contingent, 55 total events of NSSI). Nock et al. [40] studied 30 adolescents and young adults (87% female, 17.3 ± 1.9 years) with NSSI thoughts in the past 2 weeks (113.4 ± 174.9 episodes of NSSI in the past year) using EMA (14-day period, average 17.2 ± 5.3 days, 2 prompts per day, 104 incidents of NSSI). In a recent study, reporting on an overlapping sample [41], Vansteelandt et al. [42] studied *n* = 32 patients (84% female, 28 ± 9 years) with BPD of which *n* = 31 were included in the EMA analyses (8 days, 10  random prompts per day, mean interval 80 min).

Reviewing the existing literature reveals that existing studies predominantly focused on adults. Only studies by Nock et al. [40], Kranzler et al. [43], Andrews et al. [33, 34], and Armey et al. [37] included adolescents. Armey et al. included college students with lifetime NSSI and relied on a relatively small number of actual NSSI incidents (*n* = 17). The study by Nock et al. [40] included adolescents with high-frequent NSSI in the past year and analyses were based on a reasonable number of incidents (*n* = 104), however, the sampling frequency (two prompts per day) was too low to address immediate changes in affect preceding or following NSSI. Studies by Andrews et al. [33, 34] were among the few including actual treatment seeking patients, based on *n* = 24 patients reporting a total of 52 acts of NSSI, but again EMA was based on a relative low sampling frequency (6 prompts per day). The study by Kranzler et al. [43] included *n* = 47 adolescents and young adults, monitored for two weeks, reporting a total of 145 episodes of NSSI. However, again sampling frequency was low (5 random prompts per day).

Only high-frequency EMA is capable to capture immediate effects of NSSI and acute changes in affective state preceding the behavior. Given the link between impulsivity and NSSI [44, 45] and the possibility that acts of NSSI may be spontaneously initiated, capturing these fast dynamics seems key to gain a better understanding of NSSI. Importantly, previous studies most frequently focused on affect only and not interpersonal factors such as attachment. As mentioned above, motives to engage in NSSI may be driven by intrapersonal factors (i.e., intrapersonal affect regulation) and (2) social factors (i.e., interpersonal regulation of attachment) [46]. We have previously demonstrated instability in feelings of attachment towards significant others (i.e., mother and best friend) in adolescents engaging in NSSI [47]. Therefore, alongside momentary affect, we were interested to see, how changes in feelings of momentary attachment would predict acts of NSSI.

### Aims of the study

Overcoming the limitations of existing research, we aimed to address affective and interpersonal states preceding and following NSSI in female adolescents with NSSI-disorder using hourly high-frequency sampled EMA. In line with previous findings summarized above, it was hypothesized that negative affect would be increased preceding NSSI and would be decreased following NSSI. It was hypothesized that feelings of attachment towards the mother and best friend would reveal similar patterns (i.e., decreased attachment prior to NSSI).

## Methods

Data for the present analysis were pooled among different EMA studies including female adolescents with NSSI disorder using the same EMA assessments conducted at the Department of Child and Adolescent Psychiatry, Heidelberg University. The authors assert that all procedures contributing to this work comply with the ethical standards of the relevant national and institutional committees on human experimentation and with the Helsinki Declaration of 1975, as revised in 2013. All procedures involving human subjects/patients were approved by the ethics committee of the Medical Faculty at Heidelberg University (Approval Number: S-448/2014). All participants and their legal guardians signed written informed consent prior to participation in the studies. NSSI is commonly associated with both suicidal ideation and behavior [5–9]; thus, it is important to note that all clinical participants were recruited from our outpatient program, which includes particularly rigorous crisis planning and unlimited access to all levels of care within our clinic, in the case of suicidality during data collection.

All patients underwent structured clinical interviews including the Self-Injurious Thoughts and Behavior Interview (SITBI-G) [48], the borderline personality disorder part of the German version of the Structured Clinical Interview for DSM-IV Personality Disorders (SCID-II) [49], and the Mini-International Neuropsychiatric Interview for Children and Adolescents (M.I.N.I-KID 6.0) [50].

### Ecological momentary assessment

After completing diagnostic assessments, female participants received a study smartphone with the movisensXS experience sampling app (Movisens GmbH, Karlsruhe, Germany). movisensXS enables the programming of smartphones to function as electronic diaries. All participants were instructed and trained regarding the use of the smartphones and corresponding app. EMA assessments started the weekend following the handout of smartphones. Given that smartphone use is prohibited in regional schools, assessment days were limited to weekends (Saturdays and Sundays). Depending on the study, participants carried the e-diary on one or two consecutive weekends, i.e., on a total of 2 or 4 days. The movisensXS app emitted an acoustic prompting signal according to a random time-sampling schedule on average once per hour (i.e., in 60 min intervals) with a target number of 12 assessments per subject per day. Prompting signals started at 10 am and finished regularly at 10 pm. To maximize compliance and to increase the probability to obtain 12 assessments per participant per day, participants were enabled to postpone up to 2 alarms that were amended on the respective day (prompts finishing at midnight). Each completed response was automatically time-stamped by the app. Data were assessed, uploaded and stored pseudonymized and encrypted on both devices and movisens servers. After completing the assessments participants returned the smartphones, were debriefed and financially compensated. Participants received 12 Euros for taking part in the diagnostic assessments and 1 Euro for every completed assessment, given a minimum of 50% completed prompts.

At each prompt, participants were asked a number of questions. First, participants were instructed to rate their momentary affect and current attachment towards significant others (mother and best friend). Current affective states were assessed based on a four item short-form of the Multidimensional Mood Questionnaire designed for the usage in EMA studies [51]. Participants responded to the statement “At this moment I feel…” by means of four bipolar items (two positively poled items: unwell–well, agitated–calm; and two negatively poled items: content–discontent, relaxed–tense). Participants rated each item on a visual analog scale [VAS] ranging from 0 to 100. These items have shown both very good psychometric properties and good sensitivity to change [51]. Next, participants were asked to indicate whether they engaged in dysfunctional behavior within the past hour since the preceding assessment. Participants had the option to endorse the following responses (multiple responses possible): no, high-risk behavior; cutting, scratching, burning; hitting head against the wall; sexual impulsive behavior; alcohol, drugs, pills; binge eating, vomiting; other. Next, participants were asked to indicate whether they engaged in any distracting behavior since the preceding assessment (within the past hour did you engage in any of the following activities to distract yourself). Participants had the option to endorse the following responses (multiple responses possible): no, no distraction; homework/learning; watching TV/playing video games; meeting with friends; sports; relaxing/sleeping; other/other distraction. Next, participants were asked to rate their current urge to self-injure on a VAS ranging from 0 to 100 (“At this moment, how strong is your urge to self-injure?”). Finally, to assess participants’ current interpersonal states four items addressing the momentary attachment to the participant’s mother and four items regarding the momentary attachment to the best friend (named in the first assessment during the training session) were used. Items assessing momentary interpersonal states were worded as: (1) How close do you feel to your mother/best friend right now?; (2) How important is your mother/best friend to you right now?; (3) What do you think, how close does your mother/best friend feel to you right now?; (4) What do you think, how important are you for your mother/best friend right now? Participants rated each item on VAS ranging from 0 to 100. Beyond good compliance, the respective EMA methodology showed reliability and validity in our previous studies, as evidenced by correlations of EMA-derived measures with clinical interviews [47].

### Data preprocessing and analyses

For the present analyses endorsements of *cutting, scratching,* and *burning* were recorded as events of self-injury. Any endorsement of any distracting activity was coded as distraction. Analyses focused on ± 1 h surrounding actual reports of self-injury. A composite negative affect score was created by invers scoring the two positively poled items and then calculating the mean value of the four affect items for each assessment. Possible values of the mean scores range from 0 to 100, with a higher score corresponding to a greater negative affective state. Two attachment scores (mother and best friend) were created by calculating the mean value of the respective four attachment items for each administration of the scale. Possible values of these mean scores range from 0 to 100, with higher scores corresponding to greater levels of momentary attachment. Scores were standardized to distinguish within from between effects on different levels of analyses. First, scores were *z*-standardized across all assessments (independent of subject and day) to enhance comparability of regression coefficients (termed: global-level). Second, the mean for each subject was calculated and subtracted from the standardized score (termed: subject-level). Third, the mean of each day for each subject was calculated. To achieve a pure within-subject and day predictor, the mean for each day of assessment for each subject was subtracted from the standardized score (termed: within-level).

First, multilevel mixed-effects logistic regressions with robust variance estimation predicting self-injury by the (M1) urge to self-injure, (M2) negative affect and (M3) interpersonal attachment (mother and best friend) preceding reported acts of self-injury were calculated for each within and between effect (global-, subject-, and within-level). The subject ID and day of assessment within subjects were included as random effects to control for interpersonal differences and differences between different days of assessments in all models. Concrete, the respective models predicted the likelihood of NSSI, based on ratings on the urge to self-injure, negative affect and interpersonal attachment (mother and best friend) preceding actual self-injury in contrast to ratings not preceding self-injury adjusted for differences between individuals and day of assessment. First, single models for each predictor (urge to self-inure, negative affect, attachment towards mother, attachment toward best friend) were calculated, followed a stepwise logistic regression minimizing the Bayes Information Criterion [BIC]. Predictors that survived the stepwise regression were subjected to further analyses, addressing their interaction with distracting activities in mixed-effects logistic regressions with robust variance estimation.

Second, to address consequences of self-injury on the urge to self-injure, negative affect, and interpersonal attachment (mother and best friend), mixed-effects regression with robust variance estimation were calculated predicting change in the aforementioned variables based on self-injury. Successive differences scores were calculated for the urge to self-injure, negative affect and interpersonal attachment, each between two adjacent assessments. Events of self-injury (yes/no) were included as fixed effect. Again, ID and the assessment day within ID were included as random effects to control for inter-day and inter-person variability. All analyses were conducted in Stata/SE (15.0, Stata Corp LLC, College Station, TX, USA), at an alpha level of 0.05.

## Results

### Pooled data and study sample

**Fig. 1:**
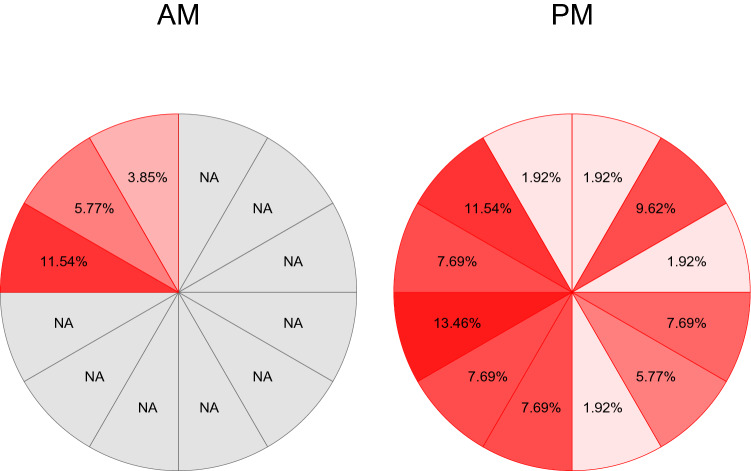
Acts of self-injury by time of day; percentage values relative to the total acts of self-injury recorded (*n* = 52); *AM* EMA was conducted from 9 AM till midday, *PM* EMA was conducted from 1 PM till midnight, *%* relative percentage of NSSI reported within the specific hour based on a total of *n* = 52 actual acts of NSSI

Data from a total of *n* = 73 female adolescents were available for analyses. Adolescents participated in various studies including the same EMA at the Department of Child and Adolescent Psychiatry in Heidelberg. Samples included case–control data that were previously published [47] and data from ongoing treatment studies, using pre-treatment assessments only. Given the event-related nature of the planned analyses, data from all subjects were pooled. Sociodemographic and clinical characteristics of the sample are provided in Table 1. On average, participants completed 3 days of EMA with an average of 9.73 (SD = 3.04) daily assessments. The entire sample comprised a total of *n* = 2131 observations (hourly assessments). Actual acts of self-injury during EMA were reported by *n* = 20 patients with an average frequency of 2.60 (SD = 2.48; range 1–11). A total of 52 acts of self-injury were available for analyses (2.44% of total observations). Acts of self-injury were reported independent of the time of day, with the majority of events occurring in the evening as illustrated in Fig. 1.

**Table 1:** Clinical and Sociodemographic Characteristics of the Sample; ICD-10 diagnoses: multiple diagnoses possible; BPD: borderline personality disorder based on SCID interview; NSSI frequency based on SITBI interview; school: after four years of elementary school the German school system branches into three types of secondary schools. The so called Hauptschule (Secondary General School which takes five years after Primary School) prepares pupils for vocational training, whereas the Realschule (Intermediate Secondary School) concludes with a general certificate of secondary education after six years. Eight years of Gymnasium provide pupils with a general university entrance qualification

*N* (female)	73 (73)
Age, mean (SD)	15.48 (1.19)
School, *n* (%)	
Hauptschule	4 (5.48)
Realschule	28 (38.36)
Gymnasium	28 (38.36)
Other	13 (17.8)
ICD-10 diagnoses, *n* (%)	
F1	21 (28.77)
F2	1 (1.37)
F3	49 (67.12)
F4	39 (53.42)
F5	11 (15.07)
F6	24 (32.88)
F9	14 (19.18)
NSSI frequency past year, mean (SD)	104.88 (117.99)
BPD, *n* (%)	41 (56.16)
BPD criteria, mean (SD)	4.68 (2.04)

### Momentary predictors of self-injury

**Fig. 2:**
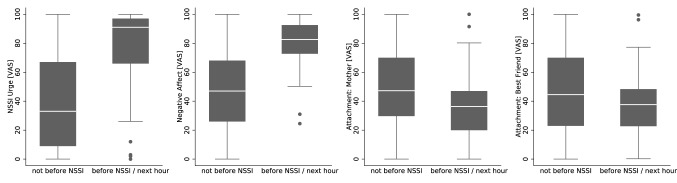
Predictors of Acts of Self-Injury; illustrated is the mean urge to self-injure (**a**), negative affect (**b**), attachment towards the mother (**c**) and best friend (**d**) before and not-before reported self-injury; *VAS* visual analogue scale, *NSSI* non-suicidal self-injury

The urge to self-injure predicted actual self-injury in the following hour on the *global* (OR: 1.63, 95% CI [1.04; 2.55]) and *subject-level* (OR: 5.99, 95% CI [2.30; 15.58]), but was not predictive on the *within-level* (OR: 3.79, 95% CI [0.78; 18.44]). Greater negative affect was associated with an increased likelihood to report self-injury in the following hour on the global (OR: 1.83, 95% CI [1.11; 3.02]), subject- (OR: 10.39, 95% CI [2.29; 47.12]), and within-level (OR: 16.42, 95% CI [4.82; 55.93]). Feelings of attachment towards the mother, were only predictive for self-injury on the subject-level (OR: 0.39, 95% CI [0.16; 0.96]), such that greater attachment was associated with a lower likelihood of actual self-injury. Feelings of attachment towards the best friend were only predictive for self-injury on the within-level (OR: 0.27, 95% CI [0.08; 0.94]) such that greater attachment was associated with a lower likelihood of self-injury. In multiple regression analyses including all predictors, only the urge to self-injure on the subject-level (OR: 3.62, 95% CI [1.10; 11.91]), negative affect on the global- (OR: 1.77, 95% CI [1.15; 2.73]) and within-level (OR: 23.11, 95% CI [1.66; 322.75]), as well as attachment towards the mother on the subject-level (OR: 0.29, 95% CI [0.12; 0.70]) were still significant after adjusting for the other predictors. Stepwise regression minimizing the BIC emphasized the urge to self-injure on the subject-level (OR: 6.76, 95% CI [2.51; 18.23]) and negative affect on the within-level (OR: 14.85, 95% CI [4.16; 53.02]) to be the best predictors of self-injury. Findings of effects on the global-level are illustrated in Fig. 2. Reported distraction by other actives had no significant effect on actual self-injury and did not moderate the effect of negative affect (interaction distraction x affect: OR = 1.31, *p* = 0.558).

### Momentary consequences of self-injury

**Fig. 3:**
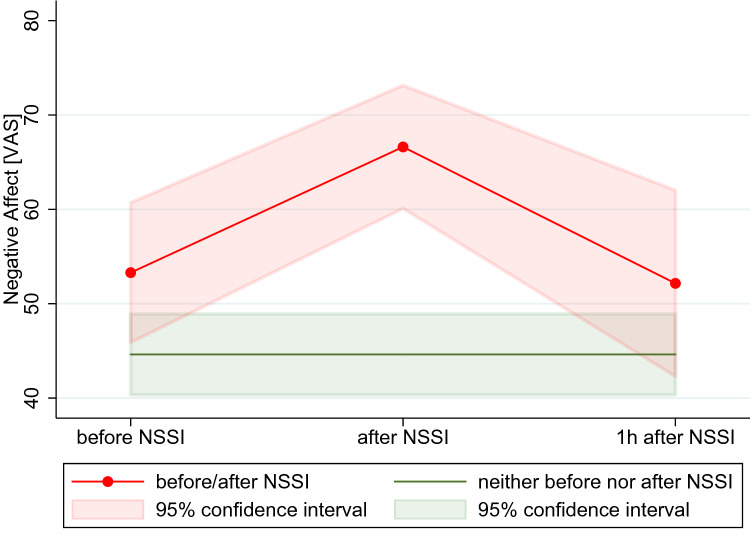
Negative affect preceding and following self-injury; mean and 95% confidence interval of negative affect before, after and 1 h after acts of self-injury; *VAS* visual analogue scale, *NSSI* non-suicidal self-injury

For a total of *n* = 34 acts of self-injury, there was sufficient non-missing data from assessments taken in the following hour to assess consequences of NSSI. There were a total of four cascades of NSSI (acts of self-injury reported in 2 consecutive hours on the same day of assessment), that were included in the analyses. Actual self-injury had no significant effect on the urge to self-injure (*χ*^2^_(1)_ = 0.82, *p* = 0.366). Interestingly, the urge to self-injure slightly increased following self-injury (Δ*z* = 0.21, SD = 1.16) compared to changes in adjacent assessment without reporting of self-injury (Δ*z* = 0.01, SD = 0.81). Self-injury resulted in significant increases in negative affect (*χ*^2^_(1)_ = 9.23, *p* = 0.002). As depicted in Fig. 3, negative affect increased (Δ*z* = 0.36, SD = 0.83) following acts of self-injury, compared to changes in adjacent assessment without reporting of self-injury (Δ*z* = − 0.005, SD = 0.89). Self-injury decreased (Δ*z* = − 0.22, SD = 0.57) the attachment towards the mother (*χ*^2^_(1)_ = 5.18, *p* = 0.016) compared to recordings with no reported self-injury (Δ*z* = − 0.002, SD = 0.48). There were no effects of self-injury on the attachment towards the best friend (*χ*^2^_(1)_ = 0.64, *p* = 0.423).

## Discussion

The present study sought to identify short-term predictors and consequences of actual self-injury, addressing affective and interpersonal states in everyday life of female adolescents engaging in NSSI. Pooling of data resulted in a sample of *n* = 73 female adolescents, of whom 20 reported a total of 52 acts of NSSI in high-frequency hourly EMA. Descriptive data from the study are in line with previous reports, suggesting that most frequently self-injury is conducted in the evening hours [52]. However, considering that EMA assessments were limited to the weekends, we found a surprisingly high frequency of NSSI early in the morning, i.e., in the hour preceding the first assessment at 10 am.

Addressing predictors of actual acts of self-injury, results illustrate that the urge to self-injure on the subject-level and negative affect on the within-level predicted subsequent NSSI. In line with previous research, negative affect was the strongest predictor of NSSI [41]—among those that were investigated (i.e., affect, urge, attachment towards the mother or best friend). Changes in interpersonal states, such as the felt attachment towards the mother and best friend, did not predict the likelihood of reporting self-injury in the following hour. An interesting finding is that distraction by other activities (i.e., homework, watching TV, meeting with friends, sports), did not change the predictive value of negative affect. Thus, it seems key to reduce negative affect in the first place. Based on our analyses, it seems plausible to hypothesize, that once negative affect is too high, distraction is unlikely to prevent acts of NSSI. In this regard, future studies, addressing the effect of, e.g., skills taught in treatment for NSSI, such as dialectical behavioral therapy (DBT), would be interesting. Evidence from such EMA studies could inform psychotherapeutic practice and guide the use of particular techniques to prevent acts of NSSI in times of heightened emotional distress.

Regarding the consequences of NSSI, again, the present study reveals that self-injury does not result in a decrease of negative affect in the following hour. This has previously been shown by an EMA study [41] with a time interval of ~ 1.33 h between assessments, conducted in *n* = 30 adult inpatients with BPD. Other studies have shown that negative affect decreases following experimentally induced pain in adolescents with NSSI [23]. Based on the present findings, it is suggested that the momentary relief is of short duration and unlike expected, NSSI actually leads to an increase of negative affect within the hour following self-injury. Thus, findings from EMA studies on adolescent NSSI using a lower sampling-frequency, illustrating a decrease in negative affect hours after actual NSSI, likely do not capture the actual increase in negative affect immediately following NSSI, but illustrate a regression to the mean after the initial increase in negative affect preceding NSSI. From a methodological viewpoint, these findings emphasize the importance of appropriate sample frequencies in EMA studies [31].

Replicating earlier findings in adults [41], the present study confirms that a potential relief from negative affect following NSSI is certainly of short duration. As previously discussed [41], this suggests that changes in affect immediately following (or during) NSSI, are capable to reinforce the behavior and that negative consequences on the time scale of hours are neglected or are not relevant concerning the maintenance of NSSI. The decrease in negative affect following self-injury, as reported by EMA studies in adults at lower sample rates (e.g., 2 h), is in line with the present findings, illustrating a return of negative affect to levels comparable to those reported before self-injury, 1–2 h after NSSI [37]. However, the present findings emphasize that this decrease in negative affect is preceded by an initial increase in negative affect following NSSI.

The study has several strengths and limitations that should be addressed. In comparison to previous studies in adolescents and adults, we used a relatively high-frequency sampling approach based on hourly EMA, report on a large sample covering a reasonable number of actual events of self-injury and included outpatients with severe NSSI-disorder. However, analyses were still limited to *n* = 20 patients reporting self-injury within EMA assessments limited to days of the weekend. Further, we only included females and findings might not generalize to male patients. We are not able to determine the actual time of self-injury (on a scale of minutes) and, accordingly, the time between self-injury and respective ratings on self-report measures. EMA studies enabling patient emitted assessments (i.e., in the event of self-injury) may help to focus on the minutes following acts of self-injury and overcome these limitations. Future studies would do well to further distinguish different qualities of negative affect in the context of NSSI. For example, it is possible that our aggregated measure of negative affect conceals differential dynamics in affect by the antagonistic states of distress and e.g., feelings of shame. As such, it is possible that NSSI decreases distress but increases shame and that such effects result in an overall increase of negative affect (umbrella term).

To conclude, the present study illustrates that the affect regulating function of NSSI in female adolescents is short-lived and that self-injury may actually increase negative affect. This finding challenges common beliefs about affect regulation in NSSI and should inform practice in clinical care (i.e., psychoeducation). Patients should be educated that the engagement in NSSI prospectively predicts an increase in negative affect. Intended effects of relieve from negative affect are at best short-lived (< 1 h) and should be traded off against longer lasting aggravations of affective states.
